# Volatile metabolomic signature of human breast cancer cell lines

**DOI:** 10.1038/srep43969

**Published:** 2017-03-03

**Authors:** Catarina L. Silva, Rosa Perestrelo, Pedro Silva, Helena Tomás, José S. Câmara

**Affiliations:** 1 CQM - Centro de Química da Madeira, Universidade da Madeira, Campus Universitário da Penteada, 9020-105 Funchal, Portugal; 2 Departamento de Química, Faculdade de Ciências Exatas e Engenharia da Universidade da Madeira, Universidade da Madeira, Campus Universitário da Penteada, 9020-105 Funchal, Portugal

## Abstract

Breast cancer (BC) remains the most prevalent oncologic pathology in women, causing huge psychological, economic and social impacts on our society. Currently, the available diagnostic tools have limited sensitivity and specificity. Metabolome analysis has emerged as a powerful tool for obtaining information about the biological processes that occur in organisms, and is a useful platform for discovering new biomarkers or make disease diagnosis using different biofluids. Volatile organic compounds (VOCs) from the headspace of cultured BC cells and normal human mammary epithelial cells, were collected by headspace solid-phase microextraction (HS-SPME) and analyzed by gas chromatography combined with mass spectrometry (GC–MS), thus defining a volatile metabolomic signature. 2-Pentanone, 2-heptanone, 3-methyl-3-buten-1-ol, ethyl acetate, ethyl propanoate and 2-methyl butanoate were detected only in cultured BC cell lines. Multivariate statistical methods were used to verify the volatomic differences between BC cell lines and normal cells in order to find a set of specific VOCs that could be associated with BC, providing comprehensive insight into VOCs as potential cancer biomarkers. The establishment of the volatile fingerprint of BC cell lines presents a powerful approach to find endogenous VOCs that could be used to improve the BC diagnostic tools and explore the associated metabolomic pathways.

Although there has been a sustained decline in mortality rates over recent decades, breast cancer (BC) continues to be the most prevalent malignancy among women worldwide and is a major cause of female deaths. A number of associated factors, including age, gender, ethnicity, lifestyle (tobacco, alcohol, diet, and lack of exercise) and genetics, such as mutations in the tumor suppressor genes BRCA1 (mutation on chromosome 17) and BRCA2 (mutation on chromosome 13), have been identified as the most common inherited causes of susceptibility to BC. The BRCA1 and BRCA2 genes encode very large proteins that are expressed in a wide variety of different tissues and are implicated in processes such as DNA repair and recombination, checkpoint control of the cell cycle, and transcription. Families with a high incidence of BC may carry mutations in one or both of these genes, or alternatively, members of these families may have similar lifestyle habits and may have been affected by similar environmental factors^1^,^2^. Furthermore, other important gene mutations related to BC development include mutations in ATM (ataxia-telangiectasia mutated), TP53 (tumor protein p53), and PTEN (phosphatase and tensin homolog deleted on chromosome ten)^3^,^4^,^5^. Most of these are rare and often do not increase the risk of BC as much as do mutations in the BRCA genes. Detection of BC at an early stage is extremely important to reduce the burden of disease because earlier detection leads to better patient outcomes, as metastatic states are avoided^6^,^7^. Currently, there is no single screening test that is totally reliable, and a number of tests can be combined to help detect early stage BC. Current screening techniques include self-examination for lumps or nodes, mammography, ultrasound, magnetic resonance imaging and biopsy using a fine needle or similar instrument to aspirate or otherwise remove a sample of fluid or cells from any suspicious lump or node for microscopic examination^8^. These methods are generally invasive, time-consuming and require special medical skills. Therefore, there is a need for non-invasive, accurate and rapid screening tests for early detection. In this context, it is useful to identify volatile organic compounds (VOCs) that may prevent or predict the occurrence of metastasis before it manifests in the patient.

Metabolomics has emerged as a powerful tool for understanding biological processes that occur in humans, and it has mostly been based on the analysis of biofluids, such as blood, saliva, or urine, to discover new cancer biomarkers or to diagnose a disease^9^.

The use of cell culture metabolomics enables both the discovery of novel biomarkers of pathological conditions and investigation of the related metabolomic pathways (Fig. 1). Many of the metabolic processes in the body, such as lipid peroxidation, energy metabolism through glycolysis and amino acid catabolism are common to all living cells^9^. It is believed that some metabolic pathways might be up- or down-regulated in cancer cells and, therefore, metabolome analysis may reveal differences between biological samples based on metabolic profiles or fingerprints. Indeed, cancer cells have an altered metabolism compared with normal cells that may lead to the production of specific compounds^10^. In recent years, several studies have reported the analysis of cancerous cell lines to find potential cancer biomarkers^11^,^12^,^13^,^14^. The most recent techniques include the use of nanomaterial-based sensors^11^, electrochemical sensors^15^, or thermal desorption coupled with gas chromatography mass spectrometry^16^. However, most of these techniques are expensive and time-consuming. In this work, solid-phase microextraction in headspace mode (HS-SPME), which was developed by the Pawliszyn group^17^ in the early 90’s and consists of a fiber coated with different polymers extracting a wide range of chemical compounds, was selected as an extraction technique. This technique is superior to other extraction techniques, in that it is rapid, easy to use, sensitive and does not require a concentration step before analysis.

In this study, a comparative analysis of the volatile metabolomic signature of BC cell lines (T-47D, MDA-MB-231, MCF-7) and normal human mammary epithelial cells (HMEC), was carried out, in order to identify BC-specific VOCs and to identify a set of biomarkers that could hopefully be correlated with VOCs released *in vivo* by BC cells. This finding will improve the knowledge about the origin of VOCs and providing comprehensive information as potential BC biomarkers. This strategy can help reveal novel BC biomarkers that might expand the current understanding of this multi-factorial disease. The GC–qMS analyses allow specific identification of VOCs, while multivariate statistical analysis is able to differentiate and discriminate oncologic from normal cells providing proof-of-principle for the detection of different volatile metabolomic patterns in target cells.

## Results and Discussion

VOCs associated with normal breast cells (HMEC) and BC cell lines (T-47D, MDA-MB-231 and MCF-7 cells) were investigated. The cell lines for the present study were chosen based on their different molecular characteristics (Supplementary Table S1): namely, the expression of the estrogen receptor (ER), the progesterone receptor (PR), and the human epidermal growth factor receptor 2 (HER2). It is well known that BC is heterogeneous and that its prognosis and treatment depends on the molecular subtype of the cancer cells. The VOCs arising from the cellular metabolism were studied using HS-SPME/GC-MS: (a) by direct analysis of the headspace of the culture flasks after cell growth (these results are hereinafter designated as “Cells”); and (b) by analysis of the volatile metabolites from the culture media at different pH values. From the analysis of chromatograms, it was possible to identify 60 VOCs belonging to distinct chemical groups, namely, alkanes, aldehydes, ketones, acids, alcohols and benzene derivatives.

### VOC signature of BC cell lines and breast normal cells

**Figure 2:**
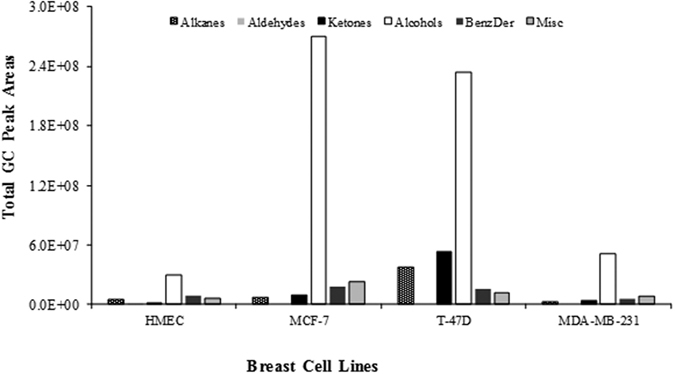
Distribution of VOCs identified in cultured breast cell lines.

We identified twenty-six VOCs belonging to several chemical groups (Fig. 2): namely, alkanes, aldehydes, ketones, acids, alcohols and benzene derivatives (Table 1).

From these VOCs, 13 were found to be common in all studied breast cells (both normal and cancerous), 5 were present only in normal breast cells (HMEC), and 2 compounds were identified only in the MCF-7 breast cell line.

As can be observed in Fig. 2, the MCF-7 cell line demonstrated the most complex volatile metabolomic signature in terms of number of the identified VOCs and total GC peak areas compared with the other cell lines. Moreover, for all BC cell lines, the major chemical group identified was the higher alcohols, represented mainly by 2-ethyl-1-hexanol and cyclohexanol. These VOCs have already been reported in previous studies using BC cell lines^18^,^19^ and in urine^20^,^21^ from cancer patients. It is believed that their endogenous origin is as hydrocarbon metabolism byproducts^22^,^23^. The obtained data indicated that the levels of both VOCs (2-ethyl-1-hexanol and cyclohexanol) were higher in all investigated BC cells than in normal cells (HMEC). This might be due to the production of lipid peroxidation biomarkers with hydroxylase that are mediated by cytochrome P450^12^,^18^.

Similar results were reported by Peled and collaborators^24^ when studying genetic mutations in lung cancer cells, and by Davies and collaborators^25^, who compared the volatile profile from the headspace of lung cancer cells with genetic mutations in TP53 and KRAS. Most of the identified VOCs were common to all BC cell lines and normal human mammary epithelial cells, but six of them, 2-pentanone, 2-heptanone, 3-methyl-3-buten-1-ol, ethyl acetate, ethyl propanoate, and 2-methyl butanoate, were detected only in BC cell lines. This finding justifies a more detailed investigation to evaluate of these six VOCs as BC biomarkers.

### The influence of pH on the VOCs identified from culture media

**Figure 3:**
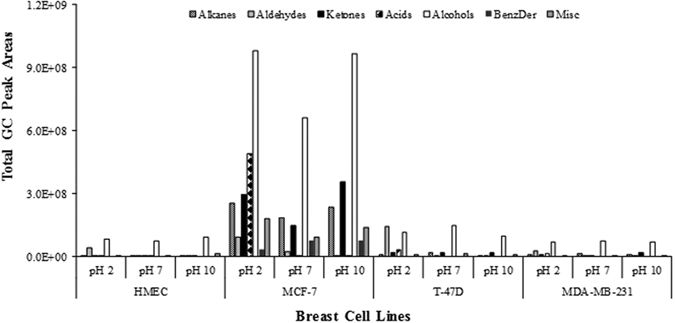
Distribution by chemical families of VOCs identified in culture media at different pH conditions of breast cell lines.

The pH is one of the parameters that influences the extraction efficiency of VOCs and therefore it is required an optimization step. This was accomplished by the assessment of volatiles from culture media at different pH. We evaluated the effect of pH on the volatile signature obtained from culture media. At pH 2, the MCF-7 cells had the highest total GC peak area with acids (hexanoic acid, octanoic acid and 2-ethyl-hexanoic acid) being the most dominant chemical group. Aldehydes (benzaldehyde and 3,4-dimethyl-benzaldehyde) were the most predominant chemical group in the T-47D and MDA-MB-231 cells (Fig. 3; Supplementary Table S2). At pH 7, for MCF-7 cells, alkanes, ketones and alcohols were found the dominant chemical groups, which were represented by dodecane, 2-heptanone, and 2-ethyl-1-hexanol. For the other breast target cells alcohols (cyclohexanol) were the most representative chemical group. Finally, at pH 10 the main chemical group identified for MCF-7 cells were alkanes, ketones and alcohols (2-ethoxy-2-methyl-propane, acetone and 2-ethyl-1-hexanol). For T-47D and MDA-MB-231 cells, alcohols represented by cyclohexanol, presented the major contribution. As previously mentioned, it is believed that cancer cells have altered metabolisms leading to different volatile metabolomic patterns. This was observed in our study, where we identified some differences between BC cell lines and normal cells (Supplementary Table S2). Several VOCs were found to be common in all breast cell lines for all conditions, including, 2-ethoxy-2-methyl-propane, acetone, 2-methyl-2-propanol, cyclohexanol, 1,3-bis(1,1-dimethylethyl)-benzene and 2-ethyl-1-hexanol which had higher levels in BC cells. Ethyl acetate was only present in the T-47D cell line (Supplementary Table S2). Kwak and collaborators^26^ described a similar study using melanoma cells and identified higher concentrations of acetone in cancer cells. The metabolomic origin of most VOCs is still unknown, as they rely on a variety of endogenous pathways and exogenous sources. Huang and collaborators^18^ reported that cyclohexanol and 2-ethyl-1-hexanol had lower concentrations in BC cells and suggested that they were generated by endogenous hydrocarbon metabolism. Hydrocarbons can be metabolized to aldehydes or ketones in the body via alcohol dehydrogenase (ADH) and cytochrome P450 activities^12^. The higher activity of cytochrome P450 may explain why BC cell lines have less cyclohexanol than normal breast cells^18^. It can also induce a variety of biological responses, including the biotransformation of alkanes, alkenes and aromatic compounds^27^. Furthermore, Philips *et al*. suggested that breast diseases are associated with increases in oxidative stress and a higher activity of P450^28^. Nevertheless, 2-ethyl-1-hexanol was found at higher levels in BC cells than in to normal cells. According to the human metabolome database, 2-ethyl-1-hexanol is involved in cell signaling, membrane integrity/stability and energy storage and it was also detected in lung cancer cell lines^16^ at increased levels when compared with the medium. At pH 10, the levels for most of the VOCs were higher in BC cells than in normal breast cells, including those of acetone, 2-pentanone, cyclohexanol, 2-ethyl-1-hexanol and acetophenone.

### PCA and PLS-DA analyses of VOCs

To verify the significance of the identified VOCs from the headspaces of the culture flasks and the cell culture media at different pH conditions, a *one-way* ANOVA test was applied to analyze the data matrix. From the identified VOCs, a total of 23 (from cultured flask headspace), 52 (from culture media at pH 2), 34 (from the culture media at pH 7) and 43 (from the culture media at pH 10) showed significant differences (*p* < 0.05) (Supplementary Fig. S1).

**Figure 4:**
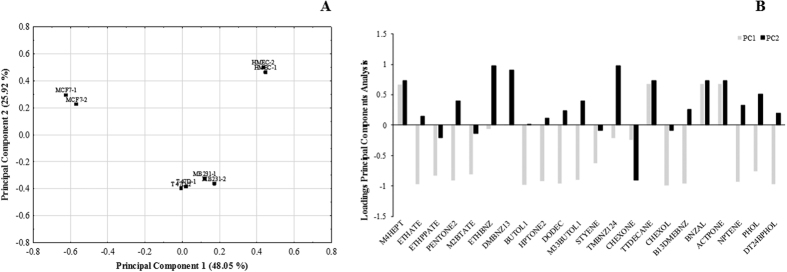
(**A**) Separation of breast cancer cell lines and normal cells based on PCA scores scatter plot and (**B**) Line plot of principal component values obtained using selected compounds by significance of *one-way* ANOVA (*p* < 0.05) obtained from the analysis of 4 types of breast cell lines.

Principal component analysis (PCA) was performed for each condition to identify variables to differentiate the VOCs pattern of the HMEC cells from those of the BC cell lines (MCF-7, T-47D and MDA-MB-231 cells), and from the VOCs patterns obtained from the culture media at different pH values (Fig. 4). The statistical data summary of PCAs are described in Supplementary Table S3. The differentiation between the above conditions was shown as the loading scores plot of the two principal components of the PCA. The PCA analysis is an unsupervised projection method used to visualize the dataset that displays the similarities and differences between groups and, in this case, demonstrated that the variables (scaled by standard deviation) used were sufficient to describe subsets with similar characteristics.

These results demonstrated that the scores from the cancer cell lines and those from the normal breast cells exhibited separate trends in the plots. Figure 4A shows the loading scatterplots of the PCA obtained from the analysis of the VOCs in the headspace of cultured flasks. It can be observed that 3 groups were formed, where HMEC cells was clearly differentiated from BC cell lines, which showed greater differentiation from MCF-7 cell lines across the PC1 and from T-47D and MDA-MB-231 across the PC2. Interestingly, BC cell lines formed two separated groups according molecular subtype (luminal A versus triple negative). However, no differentiation was achieved between T-47D and MDA-MB-231 cell lines, which formed a single group, perhaps this grouping of the two cell lines might be due to the fact that they have similar molecular characteristics. The variables that explain the differentiation between cell lines are represented in Fig. 4B. The PCs values of MCF-7 cell lines were influenced by most of variables used in this test. On the other hand, PCs values of T-47D and MDA-MB-231 cell lines were highly influenced by cyclohexanone, 1,2,4-trimethylbenzene, ethylbenzene and 1,3-dimethylbenzene for PC1, and by cyclohexanone for PC2. The HMEC cells were influenced by 4-methyl-heptane, tetradecane, benzaldehyde and acetophenone for PC1 values, and by 1,2,4-trimethylbenzene, ethylbenzene, 1,3-dimethylbenzene and phenol for PC2 values.

Concerning the other tested conditions, the loading scatterplots of the PCA obtained from the analysis of VOCs from cell culture media at pH 2, pH 7 and pH 10, and respective influence of variables, are showed in Supplementary Fig. S2. Surprisingly, four groups were formed encompassing all breast cell lines in study under pH 2 and pH 7, where MCF-7 was differentiated from other cell lines mainly across PC1, while HMEC, T-47D and MDA-MB-231 were differentiated from each other through PC2.

**Figure 5:**
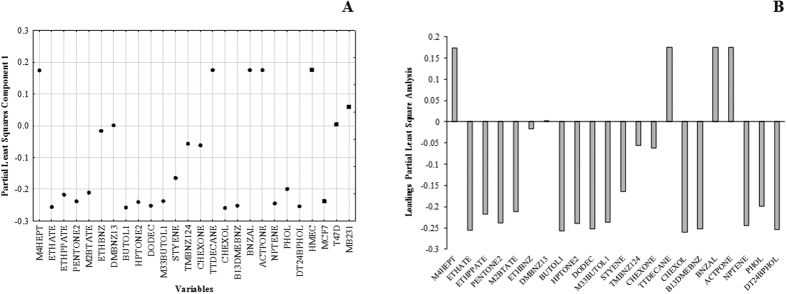
(**A**) Partial Least Square Analysis (PLS) scatter plot and (**B**) Line plot of selected compounds by significance of *one-way* ANOVA (*p* < 0.05) obtained from the analysis of 4 types of breast cell lines using cultured headspace analysis.

Under pH 10, the pattern of differentiation between cell lines is similar to obtained for headspace, in which 3 groups (HMEC, T-47D/MDA-MB-231 and MCF-7) were formed. The differentiation between cell lines obtained under pH 2 and pH 7 may be due to the alterations of molecular components released under more acidic conditions than those normally present in the culture medium (pH 7.3). However, for differentiation and discrimination between normal breast cell lines and oncological breast cell lines based on the VOCs emitted as close to reality as possible in human cell tissues, partial least squares analysis (PLS) and linear discriminant analysis (LDA) were performed only with data from headspace of cell cultures. The statistical data summary of PLS and LDA are described in Supplementary Tables S5 and S6, respectively. Sample classification by PLS showed that the differentiation between cell lines was explained through one single component. PLS loading lineplot are presented in Fig. 5, which can be observed four centroids corresponding to each cell lines.

Similar to obtained in the PCA, HMEC centroid was clearly differentiated from oncologic breast cell lines, and MCF-7 (triple negative type) was distinguished from two luminal A type cell lines. On the other hand, T-47D and MDA-MB-231 remain very close to each other, which PLS values was 0.0588 and 0.0037, respectively. Regarding the influence of variables on PLS values of cell lines, HMEC was highly influenced by 4-methyl-heptane, tetradecane, benzaldehyde and acetophenone, T-47D and MDA-MB-231 were influenced by cyclohexanone, 1,2,4-trimethylbenzene, ethylbenzene and 1,3-dimethylbenzene, and MCF-7 was influenced by the remaining VOCs.

**Figure 6:**
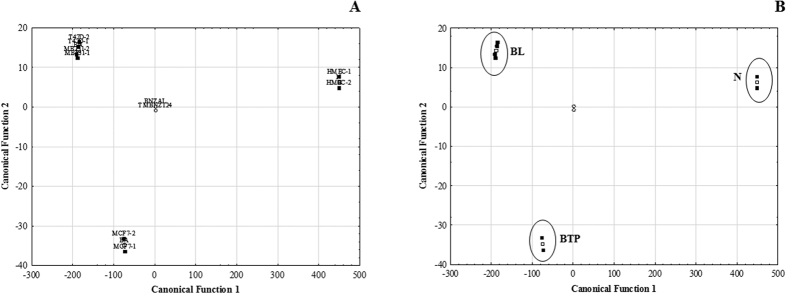
(**A**) Linear discriminant analysis (LDA) scatter plot of cultured headspace from breast cell samples (**B**) Classification of breast cells according to the canonical discriminant functions. Legend: BL-breast luminal; N- normal; BTN- breast triple negative.

The linear discriminant analysis (LDA) was applied as a supervised pattern recognition method in order to discriminate statistically the cell lines under study, where samples were grouped according to molecular type as follows: N (HMEC), BL (T-47D and MDA-MB-231) and BTN (MCF-7). The LDA scatterplot of cell lines classification according to canonical functions were showed in Fig. 6.

The cell lines samples formed three clearly defined groups with a classification rate of 100%. Recognition ability, calculated as the percentage of members of the data set that were correctly classified, and prediction ability, calculated as the percentage of members that were correctly classified, were 100% in all cases. After applying LDA with backward removal (p < 0.05) of variables, only two VOCs proved to be significant for discrimination between three defined previously, namely 1,2,4-trimethylbenzene and benzaldehyde. These compounds have been already identified in cancer cell studies by Brunner *et al*.^29^ using PTR-MS, by Filipiak *et al*.^16^ in lung cancer cells and Mochalski *et al*.^30^ with human hepatocellular carcinoma cells where it was observed an increase in the release of this compound. Moreover, these two VOCs appear to be promise biomarkers due to fact that achieve a successful discriminant classification of samples according to molecular type of breast cell lines, demonstrating that volatile metabolomic signature of breast cells can be a useful approach to identify potential BC biomarkers for early diagnosis of BC.

## Conclusions

This study demonstrated that HS-SPME/GC-MS is a simple, rapid, sensitive and solvent-free method that can be used to establish the volatile metabolomic patterns of normal and cancer breast cells. In addition, this study showed the potential of screening the *in vitro* VOCs associated with BC to identify potential volatile biomarkers to be used in early diagnosis. The headspace of culture media of normal and cancer cell lines was analyzed at different pH conditions. Sixty VOCs were identified as belonging to several chemical groups: namely, alkanes, aldehydes, ketones, acids, alcohols and benzene derivatives. Most of the identified VOCs are common to all BC cell lines and normal human mammary epithelial cells, but six of them, 2-pentanone, 2-heptanone, 3-methyl-3-buten-1-ol, ethyl acetate, ethyl propanoate, and 2-methyl butanoate, were detected only in the headspace of cancer cell lines. Multivariate statistical data obtained in this study revealed that combining *in vitro* assays with HS-SPME/GC-MS is a useful strategy to differentiate and discriminate the volatile metabolomic signature of normal cells and BC cell lines according to molecular type, thus contributing to the discovery of novel biomarkers of BC and investigations of the related metabolomic pathways, and thereby improving the diagnostic tools for BC.

## Methods

### Materials and reagents

Phosphate buffer saline (PBS) was purchased from Sigma-Aldrich (St. Louis, MO, USA), sodium chloride was obtained from Panreac (Barcelona, Spain), the SPME holder for manual sampling of SPME fiber [50/30 μm divinylbenzene/carboxen/polydimethylsiloxane (DVB/CAR/PDMS)] and the glass vials were purchased from Supelco (Bellefonte, PA, USA). The SPME fiber was conditioned according to manufacturer’s instructions. Before each daily analysis, the fiber was conditioned for 10 min in the injector port to prevent carryover. T75 glass flasks were purchased from Ningbo (Ja-Hely Technology, China).

### Cell lines and cultivation conditions

The human breast adenocarcinoma cell line MCF-7 and human breast carcinoma cell lines T-47D and MDA-MB-231 were purchased from the Leibniz Institute DSMZ-German Collection of Microorganisms and Cell Cultures (Braunschweig, Germany). MCF-7 was grown in 90% RPMI 1640 (Life technologies, Gibco^®^) supplemented with 15% fetal bovine serum (FBS, Life technologies, Gibco^®^), 1% Antibiotic-Antimycotic solution (AA, Life technologies, Gibco^®^), 1% MEM Non-Essential Amino Acids solution (Life technologies, Gibco^®^), 1 mM sodium pyruvate (Sigma-Aldrich, St. Louis, MO, USA) and 10 μg/mL human insulin (Sigma-Aldrich, St. Louis, MO, USA); T-47D cell line was grown in 85% RPMI 1640 supplemented with 15% fetal bovine serum (FBS), 1% Antibiotic-Antimycotic solution and 10 μg/mL human insulin, while the MDA-MB-231 cell line was grown in 85% RPMI 1640 supplemented with 15% fetal bovine serum (FBS) and 1% Antibiotic-Antimycotic solution. Human mammary epithelial cells (HMEC) were purchased from Life technologies (Gibco^®^) and grown in HUMEC serum-free medium supplemented with 20 μg/mL of Antibiotic-Antimycotic solution (Life technologies, Gibco^®^). All cells were incubated in a humidified atmosphere containing 5% CO_2_ and 95% air at 37 °C. Culture media was changed every 2 days and the cultures were passaged with 0.25% trypsin-EDTA (Life technologies, Gibco^®^) when 80% of confluence was achieved.

### VOC extraction from cell cultures

To extract VOCs from cell cultures, glass flasks were treated with collagen to promote cell adherence. Briefly, the glass flasks were covered with a collagen solution (0.2 mg/mL) for 30 min and then washed with PBS (3 times). The cells were then cultured in the T75 flasks for 48 h. After this period, volatile metabolites were extracted using a 50/30 μm divinylbenzene/carboxen/polydimethylsiloxane (DVB/CAR/PDMS) SPME fiber exposed in the headspace of the flasks for 45 min at 37 °C, followed by injection into the GC injection port for 10 min to allow the desorption of VOCs from the fiber. After these extractions, cell-free aliquots were collected from the flasks holding 10 mL of the culture medium with growing cells. They were centrifuged to remove any suspended cells, and then 1 mL aliquots were adjusted to pH 2, 7 or 10 with 1 M HCl or 1 M NaOH^26^. After the addition of 200 mg NaCl and subsequent stirring (0.5 mm × 0.1 mm bar) at 800 rpm, the vials were capped with PTFE septa through which the SPME fiber was inserted in the headspace of the vial and placed in a thermostatic bath at 37 °C for 45 min. After this, the fiber was withdrawn into the needle and injected in the GC port (250 °C) over 10 min, when the analytes were thermally desorbed and transferred to the analytical column. Control headspace samples were also collected from flasks containing only empty media treated with the same incubation conditions to determine the contribution to the background. The analyses were performed in triplicate.

### GC-MS analysis

VOCs in the headspace were analyzed using an Agilent Technologies 6890 N Network gas chromatograph system (Palo Alto, CA, USA) equipped with a BP-20 fused silica column (60 m × 0.25 mm I.D. × 0.25 μm film thickness, SGE, Dortmund, Germany) interfaced with an Agilent 5975 quadrupole inert mass selective detector. The following oven temperature profile was set: (a) 5 min at 45 °C; (b) increase temperature until 150 °C, at a rate of 2 °C min^−1^ (hold for 10 min); (c) 150 °C for 10 min; (d) increase temperature until 220 °C, at a rate of 7 °C min^−1^; and (e) 220 °C for 10 min. Column flow was constant at 1.0 mL/min using helium (He, N60, Air Liquide, Portugal) as the carrier gas. The injection port was maintained at 250 °C and operated in the splitless mode. Regarding MS analyses, the operating temperatures of the transfer line, quadrupole and ionization source were 270, 150 and 230 °C, respectively. The electron impact mass spectra were recorded at 70 eV and the ionization current was 10 μA, and data acquisition was performed in scan mode (30–200 *m/z*). The identification of metabolites was performed comparing mass spectra with the Agilent MS ChemStation Software (Palo Alto, CA, USA) equipped with the NIST05 mass spectral library with a similarity threshold higher than 80%, or with commercially standards when available. All experiments were performed in triplicate and the results were expressed as the mean ± standard deviation.

### Statistical analysis

Statistical tests were performed using the StatSoft STATISTICA 12.0 (2013) software (Tulsa, OK, USA). Differences in VOCs between groups were tested with *one-way* ANOVA, and *p* < 0.05 was considered as statistically significant. PCA, PLS and LDA were carried out on VOCs selected by *ANOVA* to evaluate differences among the studied groups. PCA was performed in order to obtain differentiation between samples under study without classification and PLS was used as a supervised linear pattern recognition algorithm for data classification of samples. PCA and PLS were performed through variables values scale by unit standard deviation with convergence criterion (0.0001) and leave-one-out cross validation for accuracy confirmation. For LDA analysis a backward selection method was used with a *p* < 0.05 through Wilks test. For cross validation a leave-one-out strategy was used.

## Additional Information

**How to cite this article**: Silva, C. L. *et al*. Volatile metabolomic signature of human breast cancer cell lines. *Sci. Rep.* **7**, 43969; doi: 10.1038/srep43969 (2017).

**Publisher's note:** Springer Nature remains neutral with regard to jurisdictional claims in published maps and institutional affiliations.

## Supplementary Material

Supplementary Material

## Figures and Tables

**Figure 1:**
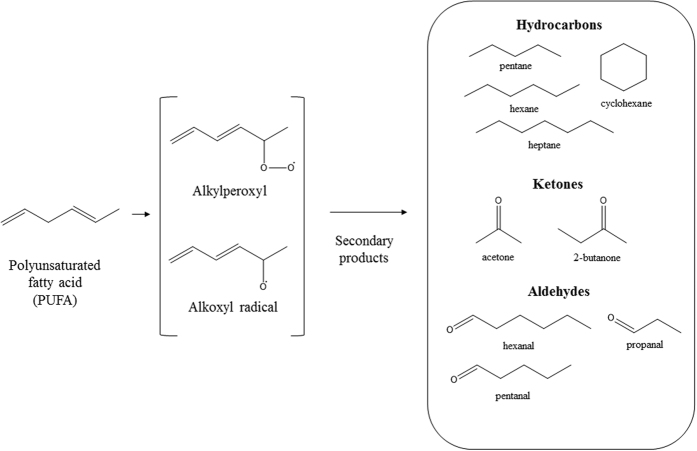
Formation of some intermediate products of lipid peroxidation. Adapted from ref. 22.

**Table 1:** Identification of VOCs from investigated human mammary epithelial cells and human BC cell lines by HS-SPME/GC-MS.

Peak no.	RT (min)	Abbreviation	Ion	VOC	HMEC (×10^5^)	T-47D (×10^5^)	MDA-MB-231 (×10^5^)	MCF-7 (×10^5^)
2	4.356	HEXA	57	hexane	0.44	—	—	—
4	5.127	M4HEPT	43, 70	4-methyl-heptane	4.83	—	—	—
5	5.739	ACTONE	43, 58	acetone	9.24	501.87	10.82	22.00
6	6.843	ETHATE	43, 61	ethyl acetate	—	8.56	3.16	25.55
8	8.716	ETHPPATE	57, 44	ethyl propanoate	—	18.11	2.17	19.66
9	9.337	PENTONE2	43, 86	2-pentanone	—	—	—	22.74
10	9.994	DECA	57, 44	decane	3.36	354.35	4.28	12.80
12	12.341	M2BTATE	57	2-methyl butanoate	—	12.83	—	13.88
17	15.865	ETHBNZ	91, 106	ethylbenzene	12.1	4.02	2.38	10.23
18	16.646	DMBNZ13	91, 106	1,3-dimethylbenzene	6.32	3.81	2.30	5.23
19	17.395	BUTOL1	56, 41	1-butanol	1.21	2.84	2.88	5.94
20	19.379	HPTONE2	43, 58	2-heptanone	—	4.96	16.46	40.00
22	20.157	DODEC	57, 43, 71	dodecane	14.19	21.29	19.93	54.42
25	23.765	M33BUTOL1	41, 56	3-methyl-3-buten-1-ol	—	—	—	3.93
26	24.155	STYENE	104, 78	styrene	64.50	139.81	39.93	125.93
27	25.511	TMBNZ124	105	1,2,4-trimethylbenzene	3.88	—	—	3.92
29	26.207	CHEXONE	55, 42, 98	cyclohexanone	2.11	20.23	12.83	8.99
32	33.450	TTDECANE	57	tetradecane	21.92	—	—	—
33	33.598	CHEXOL	57, 82	cyclohexanol	—	99.42	74.31	210.4
34	35.176	B13DMEBNZ	175, 190	1,3-bis(1,1-dimethylethyl)-benzene	4.56	9.42	7.22	31.67
36	39.305	E2HEXOL1	57, 43	2-ethyl-1-hexanol	28.32	950.78	184.81	1803.82
38	41.341	BNZAL	106, 77	benzaldehyde	2.81	—	—	—
45	48.761	ACTPONE	105, 77	acetophenone	4.44	—	—	—
47	53.752	NPTENE	128	naphthalene	3.60	3.80	3.70	5.61
54	70.775	PHOL	94	phenol	3.93	3.35	3.96	5.48
60	79.610	DT24BPHOL	191	2,4-di-*tert*-butylphenol	51.57	73.53	71.04	160.00
