# Supplementary material for: Volatile metabolomic signature of human breast cancer cell lines
Source: Sci Rep. 2017 Mar 3;7:43969. doi: 10.1038/srep43969 (PMC5335623; doi:10.1038/srep43969)
Supplement: Supplementary Material [file srep43969-s1.doc]

**Volatile metabolomic signature of human breast cancer cell lines**

**Catarina L. Silva^1^, Rosa Perestrelo^1^, Pedro Silva^1^, Helena Tomás^1,2^, José S. Câmara^1,2^,***

*^1^ CQM - Centro de Química da Madeira, Universidade da Madeira, Campus Universitário da Penteada, 9020-105 Funchal, Portugal*

*^2^ Departamento de Química, Faculdade de Ciências Exatas e Engenharia da Universidade da Madeira, Universidade da Madeira, Campus Universitário da Penteada, 9020-105 Funchal, Portugal*

**Supplementary Figure S1.** *One-way* analysis of variance (ANOVA) test in breast cell lines in studied conditions (for numbers correspondence please see Supplementary Table S2)

**
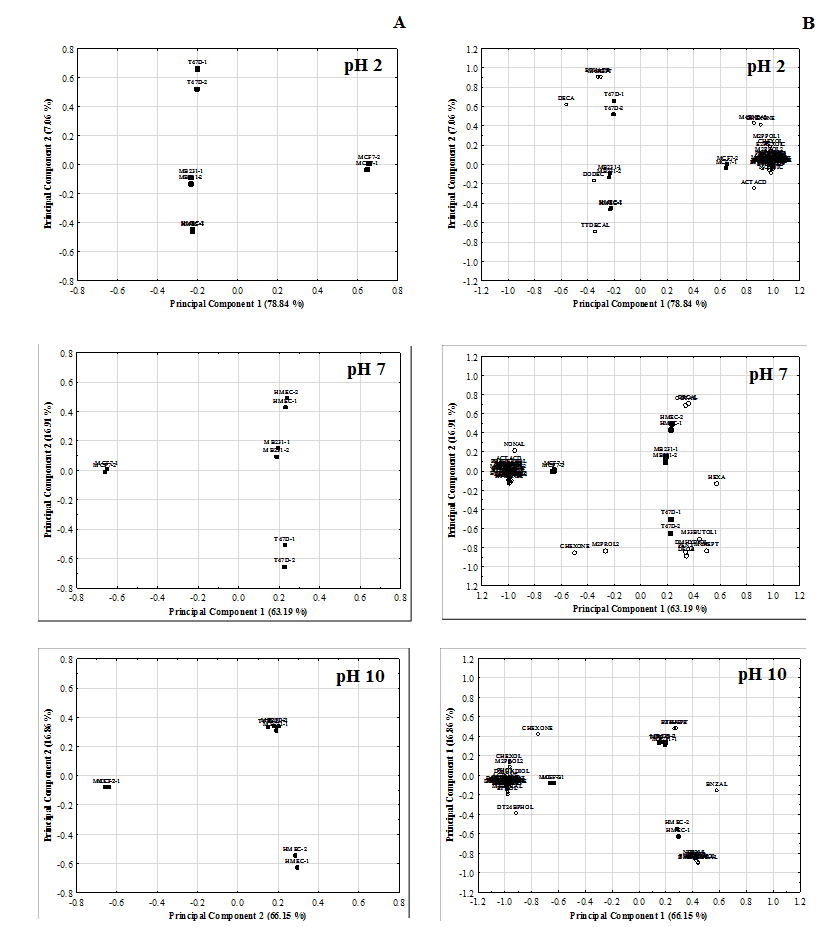
**

**Supplementary Figure S2. (A)** Loading scatterplots of variables on the PC1 × PC2 plane of culture media at different pH conditions and (**B)** Loading scatterplots of data obtained from different conditions using selected compounds by significance of *one-way* ANOVA (*p* ˂ 0.05) obtained from the analysis of 4 types of breast cell lines.

**Table S2.** Influence of pH adjustment of culture media on VOCs in the investigated human mammary epithelial cells and human BC cell lines.

| **Peak no.** | **RT (min)** | **Abbreviation** | **Ion** | **VOC** | | **HMEC (× 10^5^)** | | | | | |  | | | **T-47D (× 10^5^)** | | | | | | |  | | | **MDA-MB-231 (× 10^5^)** | | | | | | |  | | | **MCF-7 (× 10^5^)** | | | | | | |  |
| --- | --- | --- | --- | --- | --- | --- | --- | --- | --- | --- | --- | --- | --- | --- | --- | --- | --- | --- | --- | --- | --- | --- | --- | --- | --- | --- | --- | --- | --- | --- | --- | --- | --- | --- | --- | --- | --- | --- | --- | --- | --- | --- |
|  |  |  |  |  |  | **Culture media** | | | | | |  | | | **Culture media** | | | | | | |  | | | **Culture media** | | | | | | |  | | | **Culture media** | | | | | | |  |
|  |  |  |  |  |  | **pH2** | **pH7** | | **pH10** | |  | | | **pH2** | | | **pH7** | | **pH10** | |  | | | **pH2** | | | **pH7** | | **pH10** | |  | | | **pH2** | | | **pH7** | | **pH10** | |  | |
| 1 | 4.199 | PENT | 43 | | pentane | - | | - | | - | | |  | | | - | | 1.17 | | - | | |  | | | - | | - | | - | | |  | | | - | | - | | - | | |
| 2 | 4.356 | HEXA | 57 | | hexane | 5.89 | | 1.43 | | 1.53 | | |  | | | 1.33 | | 1.45 | | - | | |  | | | - | | - | | - | | |  | | | - | | - | | - | | |
| 3 | 4.478 | E2M2PROP | 59, 87 | | 2-ethoxy-2-methyl-propane | 23.52 | | 14.86 | | 26.18 | | |  | | | 102.46 | | 158.38 | | 47.22 | | |  | | | 88.73 | | 14.78 | | 10.55 | | |  | | | 2553.00 | | 1833.27 | | 2340.66 | | |
| 4 | 5.127 | M4HEPT | 43, 70 | | 4-methyl-heptane | - | | - | | - | | |  | | | 4.89 | | 24.51 | | - | | |  | | | 9.70 | | 11.86 | | 10.89 | | |  | | | - | | - | | - | | |
| 5 | 5.739 | ACTONE | 43, 58 | | acetone | 9.69 | | 6.48 | | 38.35 | | |  | | | 105.11 | | 83.7 | | 112.47 | | |  | | | 60.95 | | 39.43 | | 103.25 | | |  | | | 1175.82 | | 562.81 | | 1336.20 | | |
| 6 | 6.843 | ETHATE | 43, 61 | | ethyl acetate | - | | - | | - | | |  | | | 58.08 | | 78.00 | | 59.22 | | |  | | | - | | - | | - | | |  | | | - | | - | | - | | |
| 7 | 7.118 | M2PROL2 | 59 | | 2-methyl-2-propanol | 15.63 | | 11.07 | | 14.83 | | |  | | | 69.43 | | 79.87 | | 102.61 | | |  | | | 73.65 | | 62.40 | | 108.55 | | |  | | | 489.07 | | 66.10 | | 482.28 | | |
| 8 | 8.716 | ETHPPATE | 57, 44 | | ethyl propanoate | - | | - | | - | | |  | | | 9.01 | | 14.30 | | - | | |  | | | - | | - | | - | | |  | | | 355.78 | | 266.99 | | 20.94 | | |
| 9 | 9.337 | PENTONE2 | 43, 86 | | 2-pentanone | - | | - | | 9.17 | | |  | | | 25.48 | | 17.85 | | - | | |  | | | 11.90 | | - | | 23.70 | | |  | | | 306.59 | | 158.55 | | 319.72 | | |
| 10 | 9.994 | DECA | 57, 44 | | decane | - | | - | | - | | |  | | | 4.41 | | 4.79 | | - | | |  | | | - | | - | | - | | |  | | | - | | - | | - | | |
| 11 | 10.383 | MIKONE | 43, 58 | | methyl isobutyl ketone | 2.60 | | - | | - | | |  | | | - | | - | | - | | |  | | | - | | - | | - | | |  | | | 103.12 | | 41.07 | | 119.41 | | |
| 12 | 12.341 | M2BTATE | 57 | | 2-methyl butanoate | - | | - | | - | | |  | | | - | | 2.24 | | - | | |  | | | - | | - | | - | | |  | | | 49.51 | | 48.21 | | 26.78 | | |
| 13 | 13.381 | DMDSD | 94, 79 | | dimethyl disulfide | 0.56 | | - | | - | | |  | | | 2.34 | | - | | - | | |  | | | 2.61 | | - | | - | | |  | | | 105.27 | | - | | - | | |
| 14 | 13.721 | HEXAL | 44, 56 | | hexanal | - | | - | | 14.00 | | |  | | | - | | - | | - | | |  | | | - | | - | | - | | |  | | | 22.55 | | - | | - | | |
| 15 | 14.698 | M2PPOL1 | 43 | | 2-methyl-1-propanol | - | | - | | - | | |  | | | 12.15 | | 17.58 | | - | | |  | | | - | | - | | - | | |  | | | 45.01 | | 99.58 | | 47.43 | | |
| 16 | 15.097 | M2PPOL2 | 59, 87 | | 2-methyl-2-pentanol | - | | - | | - | | |  | | | - | | 9.01 | | 20.66 | | |  | | | - | | - | | 12.19 | | |  | | | 115.01 | | 217.07 | | 19.07 | | |
| 17 | 15.865 | ETHBNZ | 91, 106 | | ethylbenzene | - | | - | | 0.31 | | |  | | | - | | - | | - | | |  | | | - | | - | | - | | |  | | | 17.63 | | 102.18 | | 14.76 | | |
| 18 | 16.646 | DMBNZ13 | 91, 106 | | 1,3-dimethylbenzene | - | | - | | 0.48 | | |  | | | - | | - | | - | | |  | | | - | | - | | - | | |  | | | 19.61 | | 8.18 | | 18.48 | | |
| 19 | 17.395 | BUTOL1 | 56, 41 | | 1-butanol | - | | - | | 2.14 | | |  | | | 8.82 | | 11.84 | | - | | |  | | | 3.94 | | - | | - | | |  | | | 116.63 | | 40.37 | | 81.78 | | |
| 20 | 19.379 | HPTONE2 | 43, 58 | | 2-heptanone | - | | - | | 9.35 | | |  | | | 18.48 | | 25.19 | | 19.26 | | |  | | | 16.17 | | 12.54 | | 15.28 | | |  | | | 248.94 | | 165.39 | | 258.014 | | |
| 21 | 19.545 | HEPTAL | 44, 70 | | heptanal | - | | - | | 11.51 | | |  | | | - | | - | | - | | |  | | | - | | - | | - | | |  | | | - | | - | | - | | |
| 23 | 20.908 | M4HPTONE2 | 43, 58 | | 4-methyl-2-heptanone | 5.95 | | 3.98 | | 9.14 | | |  | | | 30.56 | | 15.78 | | - | | |  | | | 2.19 | | 6.66 | | 26.31 | | |  | | | 941.28 | | 527.99 | | 1240.28 | | |
| 24 | 23.229 | DM46HPTONE | 43, 58 | | 4,6-dimethyl-2-heptanone | - | | - | | - | | |  | | | - | | - | | - | | |  | | | - | | - | | - | | |  | | | 111.44 | | 417.93 | | 150.15 | | |
| 25 | 23.765 | M33BUTOL1 | 41, 56 | | 3-methyl-3-buten-1-ol | - | | 0.78 | | - | | |  | | | - | | 2.79 | | - | | |  | | | - | | - | | - | | |  | | | 36.17 | | - | | 39.67 | | |
| 26 | 24.155 | STYENE | 104, 78 | | styrene | 0.50 | | 1.03 | | 0.54 | | |  | | | 1.42 | | 3.82 | | - | | |  | | | 2.12 | | - | | 1.42 | | |  | | | 200.41 | | 399.66 | | 255.03 | | |
| 28 | 25.707 | OCTAL | 43, 56 | | octanal | - | | 4.88 | | 2.55 | | |  | | | - | | - | | - | | |  | | | - | | - | | - | | |  | | | - | | - | | - | | |
| 29 | 26.207 | CHEXONE | 55, 42, 98 | | cyclohexanone | 1.67 | | 2.05 | | - | | |  | | | 33.96 | | 59.11 | | 41.35 | | |  | | | 12.54 | | 13.24 | | 15.51 | | |  | | | 71.33 | | 53.70 | | 55.28 | | |
| 30 | 29.744 | NONONE2 | 43, 58 | | 2-nonanone | - | | - | | - | | |  | | | - | | - | | - | | |  | | | - | | - | | - | | |  | | | 104.72 | | 58.72 | | 176.30 | | |
| 31 | 33.051 | NONAL | 57 | | nonanal | - | | 7.15 | | 6.98 | | |  | | | - | | - | | - | | |  | | | - | | - | | - | | |  | | | 20.87 | | 25.08 | | - | | |
| 33 | 33.598 | CHEXOL | 57, 82 | | cyclohexanol | 1.84 | | 2.23 | | 2.88 | | |  | | | 253.99 | | 447.42 | | 320.21 | | |  | | | 129.61 | | 69.81 | | 130.48 | | |  | | | 1114.75 | | 4392.86 | | 955.42 | | |
| 34 | 35.176 | B13DMEBNZ | 175, 190 | | 1,3-bis(1,1-dimethylethyl)-benzene | 0.96 | | 1.29 | | 1.16 | | |  | | | 4.93 | | 39.05 | | 6.73 | | |  | | | 5.26 | | 21.63 | | 19.65 | | |  | | | 104.18 | | 333.13 | | 483.91 | | |
| 35 | 37.455 | ACTACD | 43, 60 | | acetic acid | 26.67 | | 5.41 | | - | | |  | | | 10.53 | | - | | - | | |  | | | 76.70 | | - | | - | | |  | | | 49.40 | | 66.10 | | 58.51 | | |
| 36 | 39.305 | E2HEXOL1 | 57, 43 | | 2-ethyl-1-hexanol | 7.47 | | 8.45 | | 13.39 | | |  | | | 69.67 | | 60.28 | | 46.74 | | |  | | | 89.73 | | 43.03 | | 24.3 | | |  | | | 1266.58 | | 448.25 | | 1841.15 | | |
| 37 | 39.739 | DECAL | 57 | | decanal | - | | 16.54 | | 3.45 | | |  | | | - | | - | | - | | |  | | | - | | - | | - | | |  | | | - | | - | | - | | |
| 38 | 41.341 | BNZAL | 106, 77 | | benzaldehyde | 1.55 | | 1.45 | | 3.14 | | |  | | | 4.61 | | 4.58 | | 4.20 | | |  | | | - | | - | | - | | |  | | | 66.68 | | 43.38 | | - | | |
| 39 | 43.279 | OCTOL1 | 56, 71 | | 1-octanol | - | | - | | 0.66 | | |  | | | - | | - | | - | | |  | | | - | | - | | - | | |  | | | 33.84 | | 13.92 | | 34.46 | | |
| 40 | 44.364 | M2PPOIC | 43, 73 | | 2-methyl-propanoic acid | - | | - | | - | | |  | | | - | | - | | - | | |  | | | - | | - | | - | | |  | | | 98.73 | | - | | - | | |
| 41 | 45.845 | HXDECA | 57 | | hexadecane | - | | - | | - | | |  | | | - | | - | | - | | |  | | | - | | - | | - | | |  | | | 44.35 | | - | | - | | |
| 42 | 47.594 | H4BTOIC | 42, 56, 86 | | 4-hydroxybutanoic acid | - | | - | | 1.86 | | |  | | | - | | - | | - | | |  | | | - | | - | | - | | |  | | | 26.08 | | 54.58 | | 59.34 | | |
| 43 | 47.875 | BTOIC | 60, 73 | | butanoic acid | - | | - | | - | | |  | | | - | | - | | - | | |  | | | - | | - | | - | | |  | | | 45.99 | | - | | - | | |
| 44 | 48.532 | M4BNZAL | 91, 109 | | 4-methyl-benzaldehyde | 93.00 | | 4.75 | | 11.98 | | |  | | | 350.28 | | - | | 9.66 | | |  | | | 79.73 | | 7.98 | | 7.49 | | |  | | | 618.95 | | 182.47 | | 64.40 | | |
| 45 | 48.761 | ACTPONE | 105, 77 | | acetophenone | - | | 2.87 | | 3.54 | | |  | | | - | | - | | 13.10 | | |  | | | - | | - | | 11.45 | | |  | | | 232.13 | | 182.65 | | 258.68 | | |
| 46 | 50.248 | M2BTOIC | 74, 60 | | 2-methyl-butanoic acid | - | | - | | - | | |  | | | 77.19 | | - | | - | | |  | | | - | | - | | - | | |  | | | 1285.3 | | - | | - | | |
| 47 | 53.752 | NPTENE | 128 | | naphthalene | 0.64 | | 0.59 | | 0.50 | | |  | | | 2.44 | | 3.36 | | 2.82 | | |  | | | 1.94 | | - | | - | | |  | | | 979.25 | | 401.66 | | 825.36 | | |
| 48 | 55.504 | DMHXDIOL | 43, 59 | | 2,5-dimethyl-2,5-hexadienol | 3.10 | | 2.10 | | 4.11 | | |  | | | 10.22 | | 16.97 | | 17.09 | | |  | | | - | | - | | - | | |  | | | 695.19 | | - | | 50.33 | | |
| 49 | 57.638 | DM34BNZAL | 133, 105 | | 3,4-dimethyl-benzaldehyde | 316.76 | | - | | 1.65 | | |  | | | 1092.42 | | - | | - | | |  | | | 226.68 | | - | | - | | |  | | | 210.68 | | - | | - | | |
| 50 | 57.834 | PHE1THOL | 79, 107 | | 1-phenylethanol | - | | - | | - | | |  | | | - | | - | | - | | |  | | | - | | - | | - | | |  | | | 46.68 | | - | | 36.12 | | |
| 51 | 60.044 | HEXOIC | 60 | | hexanoic acid | - | | - | | - | | |  | | | - | | - | | - | | |  | | | 6.67 | | - | | - | | |  | | | 511.18 | | - | | - | | |
| 52 | 61.587 | BNZOL | 79, 108 | | benzyl alcohol | - | | - | | 0.61 | | |  | | | - | | - | | - | | |  | | | - | | - | | - | | |  | | | 27.63 | | - | | - | | |
| 53 | 67.300 | E2HEXOIC | 73, 88 | | 2-ethyl-hexanoic acid | - | | - | | - | | |  | | | 178.21 | | - | | - | | |  | | | 78.66 | | - | | - | | |  | | | 961.31 | | - | | - | | |
| 54 | 70.775 | PHOL | 94 | | phenol | 4.75 | | 2.96 | | 1.07 | | |  | | | 8.19 | | 3.74 | | 3.64 | | |  | | | 7.85 | | 3.90 | | 2.77 | | |  | | | 41.22 | | 30.32 | | 20.82 | | |
| 55 | 73.289 | OCTOIC | 60, 73 | | octanoic acid | 8.49 | | - | | - | | |  | | | 39.84 | | - | | - | | |  | | | - | | - | | - | | |  | | | 2952.04 | | - | | - | | |
| 56 | 76.322 | NONOIC | 60, 73 | | nonanoic acid | 14.04 | | - | | - | | |  | | | - | | - | | - | | |  | | | - | | - | | - | | |  | | | 157.16 | | - | | - | | |
| 57 | 74.932 | TTDECAL | 57 | | tetradecanal | 21.55 | | - | | - | | |  | | | - | | - | | - | | |  | | | - | | - | | - | | |  | | | - | | - | | - | | |
| 58 | 75.573 | PHY2ETHOL | 94, 138 | | 2-phenoxy-ethanol | 2.04 | | 0.92 | | 0.72 | | |  | | | 1.93 | | - | | - | | |  | | | - | | - | | - | | |  | | | 14.21 | | 23.32 | | - | | |
| 59 | 78.832 | DECOIC | 60, 73 | | *n*-decanoic acid | 10.81 | | - | | - | | |  | | | 19.30 | | - | | - | | |  | | | - | | - | | - | | |  | | | 341.11 | | - | | - | | |
| 60 | 79.610 | DT24BPHOL | 191 | | 2,4-di-*tert*-butylphenol | 15.24 | | 12.35 | | 128.76 | | |  | | | 25.09 | | 23.47 | | 16.86 | | |  | | | 32.82 | | 16.70 | | 22.15 | | |  | | | 284.22 | | 165.60 | | 408.36 | | |

**Table S3.** Statistical data summary of Principal Component Analysis.

| PCA | Components | R²X | R²X(Cumul.) | Eigenvalues | Q² | Limit | Q²(Cumul.) | Significance | Iterations |
| --- | --- | --- | --- | --- | --- | --- | --- | --- | --- |
| HS | 1 | 0.480 | 0.480 | 14.414 | 0.106 | 0.171 | 0.106 | S | 9 |
|  | 2 | 0.259 | 0.740 | 7.778 | 0.323 | 0.195 | 0.394 | S | 6 |
|  | 3 | 0.101 | 0.841 | 3.024 | 0.033 | 0.229 | 0.414 | S | 7 |
| pH2 | 1 | 0.788 | 0.788 | 47.306 | 0.721 | 0.157 | 0.721 | S | 2 |
|  | 2 | 0.071 | 0.859 | 4.234 | -0.048 | 0.181 | 0.708 | S | 12 |
|  | 3 | 0.055 | 0.914 | 3.281 | 0.072 | 0.214 | 0.729 | S | 6 |
| pH7 | 1 | 0.632 | 0.632 | 26.539 | 0.553 | 0.163 | 0.553 | S | 6 |
|  | 2 | 0.169 | 0.801 | 7.101 | 0.177 | 0.187 | 0.633 | S | 10 |
|  | 3 | 0.077 | 0.878 | 3.221 | 0.029 | 0.220 | 0.643 | S | 6 |
| pH10 | 1 | 0.662 | 0.662 | 33.737 | 0.583 | 0.160 | 0.583 | S | 6 |
|  | 2 | 0.169 | 0.830 | 8.601 | 0.277 | 0.183 | 0.699 | S | 7 |
|  | 3 | 0.064 | 0.895 | 3.289 | 0.017 | 0.216 | 0.704 | S | 7 |

**Table S4. Principal components (PC) and variable importance in projection (VIP) of PCA statistical tests.**

|  | **Headspace** | | | | **pH2** | | | **pH7** | | | | **pH10** | | |
| --- | --- | --- | --- | --- | --- | --- | --- | --- | --- | --- | --- | --- | --- | --- |
| **VOC abbreviation** | **PC1** | | **PC2** | **VIP** | **PC1** | **PC2** | **VIP** | **PC1** | **PC2** | **VIP** | **PC1** | | **PC2** | **VIP** |
| PENT | - |  | - | - | - | - | - | 0.344 | -0.854 | 0.920 | **-** | | **-** | **-** |
| HEXA | - |  | - | - | - | - | - | 0.577 | -0.134 | 0.845 | 0.423 | | -0.849 | 0.898 |
| M4HEPT | 0.668 |  | 0.729 | 0.988 | -0.301 | 0.900 | 0.998 | 0.502 | -0.839 | 0.983 | 0.274 | | 0.489 | 0.971 |
| ETHATE | -0.975 |  | 0.141 | 0.979 | -0.323 | 0.904 | 0.993 | - | - | - | 0.263 | | 0.484 | 0.901 |
| ETHPPATE | -0.831 |  | -0.204 | 0.996 | 0.999 | -0.006 | 0.998 | -0.997 | -0.049 | 1.000 | -0.992 | | -0.116 | 0.999 |
| PENTONE2 | -0.908 |  | 0.394 | 0.992 | 0.983 | 0.054 | 0.969 | -0.975 | -0.104 | 0.969 | -0.989 | | -0.108 | 0.995 |
| M2BTATE | -0.807 |  | -0.142 | 0.992 | 0.995 | -0.029 | 0.992 | -0.994 | -0.043 | 0.994 | -0.966 | | -0.113 | 0.946 |
| ETHBNZ | -0.060 |  | 0.976 | 0.991 | 0.919 | -0.037 | 0.869 | - | - | - | -0.989 | | -0.136 | 0.997 |
| DMBNZ13 | 0.007 |  | 0.900 | 0.969 | 0.989 | -0.048 | 0.998 | -0.996 | 0.000 | 0.994 | -0.983 | | -0.140 | 0.986 |
| BUTOL1 | -0.985 |  | 0.010 | 0.987 | 0.990 | 0.035 | 0.981 | - | - | - | -0.989 | | -0.141 | 0.999 |
| HPTONE2 | -0.916 |  | 0.120 | 0.996 | 0.994 | 0.038 | 0.992 | -0.990 | -0.131 | 0.999 | -0.996 | | -0.086 | 0.999 |
| DODEC | -0.963 |  | 0.241 | 0.994 | -0.352 | -0.168 | 0.988 | - | - | - | **-** | | **-** | **-** |
| M33BUTOL1 | -0.905 |  | 0.398 | 0.991 | 0.994 | -0.023 | 0.989 | 0.445 | -0.718 | 0.929 | -0.992 | | -0.116 | 0.999 |
| STYENE | -0.627 |  | -0.083 | 0.997 | 0.973 | -0.059 | 0.994 | -0.998 | -0.007 | 0.999 | -0.992 | | -0.116 | 0.999 |
| TMBNZ124 | -0.212 |  | 0.975 | 0.997 | - | - | - | - | - | - | **-** | | **-** | **-** |
| CHEXONE | -0.239 |  | -0.908 | 0.991 | 0.908 | 0.408 | 0.991 | -0.494 | -0.855 | 0.999 | -0.753 | | 0.426 | 0.885 |
| TTDECANE | 0.673 |  | 0.732 | 1.000 | - | - | - | - | - | - | **-** | | **-** | **-** |
| CHEXOL | -0.992 |  | -0.083 | 0.990 | 0.983 | 0.180 | 0.998 | - | - | - | -0.967 | | 0.136 | 0.980 |
| B13DMEBNZ | -0.961 |  | 0.258 | 0.995 | 0.998 | -0.003 | 0.999 | -0.994 | -0.097 | 0.998 | -0.992 | | -0.092 | 0.994 |
| BNZAL | 0.674 |  | 0.731 | 0.999 | 0.996 | 0.018 | 0.992 | -0.991 | -0.076 | 0.998 | 0.580 | | -0.152 | 0.994 |
| ACTPONE | 0.673 |  | 0.732 | 0.999 | 0.998 | -0.028 | 0.997 | -0.998 | 0.011 | 1.000 | -0.996 | | -0.083 | 0.999 |
| NPTENE | -0.933 |  | 0.325 | 0.982 | 0.970 | -0.031 | 0.943 | -0.996 | -0.006 | 0.994 | -0.991 | | -0.115 | 0.997 |
| PHOL | -0.759 |  | 0.514 | 0.975 | 0.997 | 0.063 | 0.999 | -0.999 | -0.021 | 0.998 | -0.997 | | -0.007 | 0.996 |
| DT24BPHOL | -0.968 |  | 0.200 | 0.986 | 0.975 | -0.030 | 0.997 | -0.997 | -0.063 | 1.000 | -0.919 | | -0.388 | 0.997 |
| E2M2PROP | - |  | - | - | 1.000 | 0.003 | 1.000 | -0.998 | -0.060 | 1.000 | -0.993 | | -0.096 | 0.996 |
| ACTONE | - |  | - | - | 0.993 | 0.050 | 0.988 | -0.991 | -0.122 | 0.999 | -0.997 | | -0.064 | 0.998 |
| M2PROL2 | - |  | - | - | 0.974 | 0.087 | 0.958 | -0.263 | -0.839 | 0.965 | **-** | | **-** | **-** |
| ETHOL | - |  | - | - | 0.996 | -0.020 | 0.999 | -0.996 | -0.030 | 0.999 | -0.980 | | -0.189 | 0.997 |
| DECA | - |  | - | - | -0.559 | 0.621 | 0.975 | 0.347 | -0.893 | 0.998 | **-** | | **-** | **-** |
| MIKONE | - |  | - | - | 0.999 | -0.044 | 1.000 | -0.999 | 0.000 | 1.000 | -0.992 | | -0.116 | 0.997 |
| DMDSD | - |  | - | - | 0.999 | -0.010 | 0.999 | - | - | - | **-** | | **-** | **-** |
| HEXAL | - |  | - | - | 0.999 | -0.026 | 0.999 | - | - | - | 0.441 | | -0.893 | 0.992 |
| M2PPOL1 | - |  | - | - | 0.953 | 0.227 | 0.963 | - | - | - | -0.986 | | -0.116 | 0.986 |
| M2PPOL2 | - |  | - | - | 0.999 | -0.026 | 1.000 | - | - | - | -0.965 | | 0.087 | 0.940 |
| M4HPTONE2 | - |  | - | - | 0.971 | 0.005 | 0.942 | -0.998 | -0.022 | 0.999 | -0.992 | | -0.113 | 0.998 |
| DM46HPTONE | - |  | - | - | 0.992 | -0.023 | 0.984 | - | - | - | -0.992 | | -0.116 | 0.999 |
| NONONE2 | - |  | - | - | 0.999 | -0.027 | 1.000 | -0.999 | 0.000 | 1.000 | -0.991 | | -0.116 | 0.996 |
| ACTACD | - |  | - | - | 0.858 | -0.245 | 0.990 | -0.994 | 0.058 | 1.000 | -0.990 | | -0.116 | 0.993 |
| E2HEXOL1 | - |  | - | - | 0.990 | -0.007 | 1.000 | -0.990 | -0.106 | 0.992 | -0.993 | | -0.105 | 0.997 |
| OCTOL1 | - |  | - | - | 0.998 | -0.028 | 0.997 | -0.999 | 0.000 | 0.999 | -0.990 | | -0.135 | 0.999 |
| M2PPOIC | - |  | - | - | 0.995 | -0.029 | 0.991 | - | - | - | **-** | | **-** | **-** |
| TTDECAL | - |  | - | - | -0.340 | -0.696 | 0.989 | - | - | - | **-** | | **-** | **-** |
| DM34BNZAL | - |  | - | - | - | - | - | - | - | - | 0.442 | | -0.897 | 0.999 |
| HEPTAL | - |  | - | - | - | - | - | - | - | - | 0.440 | | -0.892 | 0.988 |
| HXDECA | - |  | - | - | 0.999 | -0.026 | 0.999 | - | - | - | **-** | | **-** | **-** |
| H4BTOIC | - |  | - | - | 0.989 | -0.030 | 0.980 | - | - | - | -0.988 | | -0.146 | 0.998 |
| BTOIC | - |  | - | - | 0.999 | -0.027 | 0.999 | - | - | - | **-** | | **-** | **-** |
| M4BNZAL | - |  | - | - | 0.856 | 0.427 | 0.995 | -0.999 | 0.026 | 1.000 | -0.981 | | -0.172 | 0.993 |
| M2BTOIC | - |  | - | - | 0.999 | 0.028 | 0.999 | - | - | - | **-** | | **-** | **-** |
| DMHXDIOL | - |  | - | - | 1.000 | -0.017 | 1.000 | 0.385 | -0.814 | 0.942 | -0.945 | | -0.016 | 0.975 |
| PHE1THOL | - |  | - | - | 0.999 | -0.027 | 1.000 | - | - | - | -0.988 | | -0.116 | 0.990 |
| HEXOIC | - |  | - | - | 0.998 | -0.028 | 0.998 | - | - | - | **-** | | **-** | **-** |
| BNZOL | - |  | - | - | 0.997 | -0.028 | 0.995 | - | - | - | 0.442 | | -0.897 | 0.998 |
| E2HEXOIC | - |  | - | - | 0.987 | 0.142 | 0.995 | - | - | - | **-** | | **-** | **-** |
| OCTOIC | - |  | - | - | 0.999 | -0.018 | 0.998 | - | - | - | **-** | | **-** | **-** |
| NONOIC | - |  | - | - | 0.988 | -0.092 | 0.987 | - | - | - | **-** | | **-** | **-** |
| PHY2ETHOL | - |  | - | - | 0.992 | -0.004 | 1.000 | -0.996 | 0.028 | 0.997 | 0.439 | | -0.896 | 0.994 |
| DECOIC | - |  | - | - | 0.999 | 0.001 | 0.998 | - | - | - | **-** | | **-** | **-** |
| OCTAL | - |  | - | - | - | - | - | 0.344 | 0.684 | 0.934 | **-** | | **-** | **-** |
| NONAL | - |  | - | - | - | - | - | -0.949 | 0.211 | 0.998 | 0.414 | | -0.855 | 0.901 |
| DECAL | - |  | - | - | - | - | - | 0.359 | 0.703 | 0.994 | 0.426 | | -0.875 | 0.945 |
| HMEC-1 | 0.447 |  | 0.460 | - | -0.224 | -0.462 | - | 0.232 | 0.429 | - | 0.295 | | -0.628 | - |
| HMEC-2 | 0.435 |  | 0.498 | - | -0.223 | -0.450 | - | 0.238 | 0.491 | - | 0.283 | | -0.545 | - |
| MCF7-1 | -0.623 |  | 0.293 | - | 0.650 | -0.038 | - | -0.649 | 0.008 | - | -0.639 | | -0.075 | - |
| MCF7-2 | -0.566 |  | 0.225 | - | 0.659 | 0.004 | - | -0.659 | -0.008 | - | -0.661 | | -0.077 | - |
| T47D-1 | 0.022 |  | -0.385 | - | -0.198 | 0.656 | - | 0.230 | -0.507 | - | 0.201 | | 0.339 | - |
| T47D-2 | -0.006 |  | -0.401 | - | -0.200 | 0.518 | - | 0.228 | -0.655 | - | 0.150 | | 0.335 | - |
| MB231-1 | 0.121 |  | -0.326 | - | -0.232 | -0.093 | - | 0.192 | 0.149 | - | 0.191 | | 0.310 | - |
| MB231-2 | 0.171 |  | -0.363 | - | -0.233 | -0.133 | - | 0.187 | 0.092 | - | 0.179 | | 0.341 | - |

**Table S5.** Statistical data summary of Partial Least Square Analysis.

| **Components** | **R²X** | **R²X(Cumul.)** | **Eigenvalues** | **R²Y** | **R²Y(Cumul.)** | **Q²** | **Limit** | **Q²(Cumul.)** | **Significance** | **Iterations** |
| --- | --- | --- | --- | --- | --- | --- | --- | --- | --- | --- |
| 1 | 0.561557 | 0.561557 | 14.60037 | 0.142857 | 0.142857 | -0.031706 | 0.00E+00 | -0.031706 | S | 9 |
| 2 | 0.306333 | 0.867891 | 7.96463 | 0.142857 | 0.285714 | -0.052075 | 0.00E+00 | -0.085432 | NS | 7 |

**Table S6. Statistical data summary of Linear Discriminant Analysis.**

| **Function** | **Eigenvalue** | **Canonical R** | **Wilk's Lambda** | **Chi-Sqr.** | **df** | **p-value** |
| --- | --- | --- | --- | --- | --- | --- |
| 1 | 111309.5 | 0.999996 | 1.34E-08 | 81.56097 | 4.000000 | 1.11E-16 |
| 2 | 667.2 | 0.999251 | 1.50E-03 | 29.27062 | 1.000000 | 6.29E-08 |
